# The balance of expression of *PTPN22 *splice forms is significantly different in rheumatoid arthritis patients compared with controls

**DOI:** 10.1186/gm301

**Published:** 2012-01-20

**Authors:** Marcus Ronninger, Yongjing Guo, Klementy Shchetynsky, Andrew Hill, Mohsen Khademi, Tomas Olsson, Padmalatha S Reddy, Maria Seddighzadeh, James D Clark, Lih-Ling Lin, Margot O'Toole, Leonid Padyukov

**Affiliations:** 1 Department of Medicine, Rheumatology Unit, CMM L8:04, Karolinska Institutet & Karolinska Universitetssjukhuset, Stockholm, S-17176, Sweden; 2 BioTherapeutics Research and Development, Pfizer Research, 200 Cambridgepark Drive, Cambridge, MA 02140, USA; 3 Massachusetts Research Business Technologies, 35 Cambridgepark Drive, Cambridge, MA 02140, USA; 4 Department of Clinical Neurosciences, Division of Neuroimmunology, CMM L8:04, Karolinska Institutet & Karolinska Universitetssjukhuset, Stockholm, S-17176, Sweden

## Abstract

**Background:**

The R620W variant in protein tyrosine phosphatase non-receptor 22 (PTPN22) is associated with rheumatoid arthritis (RA). The *PTPN22 *gene has alternatively spliced transcripts and at least two of the splice forms have been confirmed to encode different PTPN22 (LYP) proteins, but detailed information regarding expression of these is lacking, especially with regard to autoimmune diseases.

**Methods:**

We have investigated the mRNA expression of known *PTPN22 *splice forms with TaqMan real-time PCR in relation to *ZNF592 *as an endogenous reference in peripheral blood cells from three independent cohorts with RA patients (*n *= 139) and controls (*n *= 111) of Caucasian origin. Polymorphisms in the *PTPN22 *locus (25 SNPs) and phenotypic data (gender, disease activity, ACPA and RF status) were used for analysis. Additionally, we addressed possible effects of methotrexate treatment on *PTPN22 *expression.

**Results:**

We found consistent differences in the expression of the *PTPN22 *splice forms in unstimulated peripheral blood mononuclear cells between RA patients and normal controls. This difference was more pronounced when comparing the ratio of splice forms and was not affected by methotrexate treatment.

**Conclusions:**

Our data show that RA patients and healthy controls have a shift in balance of expression of splice forms derived from the *PTPN22 *gene. This balance seems not to be caused by treatment and may be of importance during immune response due to great structural differences in the encoded PTPN22 proteins.

## Background

It is well established that rheumatoid arthritis (RA) is a heritable disease with a substantial genetic influence on the outcome. *PTPN22 *is one of the few undisputed genetic risk factors for RA that has been replicated in many Caucasian populations, and evidence for its being a true susceptibility gene is strong [1,2]. Since the discovery of the importance of PTPN22 in the function of lymphocytes [3,4], and especially after its association with different autoimmune diseases [1], several attempts have been made to explain the biological mechanism of how *PTPN22 *gene variants may influence protein activity and subsequent differences in cell function. The best-associated polymorphism, rs2476601, which affects amino acid 620, is an R to W missense polymorphism that may alter the function of the protein. Many studies have focused on this change of function and have indeed found evidence for immune regulatory effects, suggesting that cells with the disease-associated allele may have a gain of function for PTPN22 resulting in stronger negative regulation of T-cell activation [5] and B-cell activation [6]. There is, however, evidence for other common genetic variants in the locus that associate with disease independently of rs2476601 [7], although the independent effect has been questioned [8], and recently another missense variant at amino acid 263, rs33996649 (an R to Q polymorphism) has been associated with RA [9]. Also, a genetic interaction was previously described between *PTPN22 *risk allele R620W and the *HLA-DRB1 *shared epitope (SE) alleles [10,11]. Even though multiple roles for *PTPN22 *have been discovered, we are far from understanding the specific mechanism involved in the development of autoimmunity in general and, more specifically, of RA.

The *PTPN22 *gene (MIM ID *600716) encodes an 807 amino acid protein called lymphoid tyrosine phosphatase (LYP), which belongs to the proline-, glutamic acid-, serine-, and threonine-rich (PEST) group of non-receptor classical class I protein tyrosine phosphatases. *PTPN22 *expresses several splice forms, but there is surprisingly little known about the function and regulation of these. At least two of them are translated into proteins [3], LYP1 (designated here as *PTPN22*_v1) and LYP2 (designated here as *PTPN22*_v4) (see Figure S1 in Additional file 1 for a demonstration of LYP expression in several cell lines). LYP1 contains an amino-terminal PTP domain, a central region of unknown function, and a carboxy-terminal portion (approximately 200 amino acids) containing four proline-rich motifs termed P1 to P4. The alternatively spliced LYP2 has a shorter carboxyl terminus, resulting in the absence of the P2, P3, and P4 motifs [12].

We aim to describe the variance in the expression of the splice forms of *PTPN22 *in peripheral blood mononuclear cells (PBMCs) in individuals with RA and in healthy controls and in relation to genotype data from this locus and to other RA risk factors. Since the structure and, possibly, the function of these splice forms is different, this may reveal important regulatory effects of disease-associated alleles.

## Materials and methods

### Transcript identifiers for well established splice forms of *PTPN22 *

The RefSeq accession [3,13] transcript identifiers for well established splice forms of *PTPN22 *are: *PTPN22*_v1 (LYP1), National Center for Biotechnology Information (NCBI) accession [NM_015967.5]; *PTPN22*_v4 (LYP2), NCBI accession [NM_012411.3]; *PTPN22*_v3 (LYP3), NCBI accession [NM_001193431.1]; and *PTPN22*_v2, NCBI accession [NM_012411.4].

### Patients and controls

This study includes four cohorts of individuals; cohort population characteristics can be found in Table S1 in Additional file 2. In short, cohort I includes 44 RA patients and 44 controls from Sweden; cohort II includes 47 RA patients and 19 controls from Sweden; cohort III includes 48 RA patients and 48 controls from the US; and cohort IV includes 60 multiple sclerosis (MS) patients from the same geographical area as cohorts I and II. The controls for all cohorts were selected with consideration to gender, age and ethnicity for each patient group. All four cohorts are completely independent. RA patients in cohorts I and II were selected at the rheumatology clinic of the Karolinska University Hospital on two occasions and all correspond to American College of Rheumatology 1987 criteria for rheumatoid arthritis [14]. RA patients in cohort III were aged between 41 and 76 years and had a clinical diagnosis of active disease for at least 6 months, with a minimum age of onset of 16 years. All were on a stable dose of methotrexate for at least 12 weeks. Subjects with any significant health problem other than RA or non-RA related laboratory abnormalities were not included. The 48 controls, 34 females and 14 males, were selected to match the RA patients and had the same number of subjects per age group, with the age groups defined as 41 to 50, 51 to 60, 61 to 70, and over 71 years. All were confirmed to have normal hematologic, renal, and hepatic functions as determined by laboratory test results.

Information about anti-citrullinated protein antibody (ACPA) status, smoking habits and medication (for RA patients) were obtained from medical records for cohort I. Peripheral blood cell samples from MS patients in cohort IV were selected from an in-house biobank (Neurology Clinic, Karolinska University Hospital) containing specimens collected from individuals during routine neurological diagnostic work-up. The additional three controls for the methotrexate experiment were healthy volunteers not taking any ongoing medication.

Informed consent was obtained from all participants in all cohorts, in compliance with the latest version of the Helsinki Declaration. Regional ethical committees at all sites have approved the study.

### Genotyping

Genotyping for the locus in cohort I was performed by Illumina 300 K chip in our previous genome-wide association study (GWAS) and we employed a linkage disequilibrium map for the *PTPN22 *locus from genotyping of 3,000 RA cases and controls [15]. Additionally, we performed genotyping of rs2476601 for cohort I (which was in full agreement with data from the GWAS [15] and rs3789607 for cohort II using a TaqMan allelic discrimination assay. *HLA DRB1 *data were obtained and reported for this cohort previously [16].

### Cell culture and RNA extraction

The cell purification protocol and RNA extraction for cohorts I and IV and the additional three controls for the methotrexate experiment were described previously [17]. Briefly, sodium citrate vacuum tubes (Vacutainer, Becton-Dickinson, Franklin Lakes, NJ, USA) were used for collecting blood according to the protocol from the manufacturer. PBMCs were cleaned with PBS and spun down for 15 minutes at 300 g and resuspended in PBS for viable cell counting. Cells were then spun down for 10 minutes and resuspended in DMEM (Sigma, Steinhem, Germany) and 5 ×10^5 ^cells were used for the following cell cultivations. For cohort I, IFN**γ**, 50 U (Preprotech, London, UK) or corresponding volume of medium was added in cell cultures and cells were incubated for 6 h at 37°C. For the methotrexate experiment, cells were incubated in RPMI-1640 (Sigma, Ayrshire, UK) at a final concentration of 0, 1, 10, 100 or 1,000 μM methotrexate (Wyeth Lederle Nordiska AB, Solna, Sweden) for 24 h at 37°C. After incubation (cohort IV was unstimulated) cells were lysed with RLT buffer from the mini RNA prep kit and subsequently RNA extracted (RNeasy Mini, Qiagen, Hilden, Germany). For cohort II, peripheral blood was taken in PAX tubes followed by RNA extraction with the PAXgene Blood RNA kit according to the manufacturer's protocol (PreAnalytiX, Hombrechtikon, Switzerland). mRNA from cohort I, II and IV samples was stored at -80°C prior to cDNA synthesis, which was performed with an iScript cDNA synthesis kit following the manufacturer's protocol (BioRad, Hercules, CA, USA).

For cohort III, PBMCs were isolated from whole blood samples collected into cell purification tubes (Becton Dickinson) according to the manufacturer's recommendations. All samples were shipped at room temperature in a temperature-controlled box overnight from the collection site to the laboratory, cell differential counts taken, PBMCs purified according to the CPT manufacturer's instructions and cell pellets stored at -80°C pending RNA purification. RLT lysis buffer (with 1% (10 μl in 1 ml) β-mercaptoethanol) was added to frozen pellets, RNA isolated using an RNeasy Mini kit (Qiagen) and DNase treatment performed using the Qiagen RNase-free DNase kit. Phenol:chloroform (1:1) extraction was performed subsequently, and RNA was re-purified using the RNeasy Mini kit. Eluted RNA was quantified using a ND-1000 Spectrophotometer (Nanodrop, Wilmington, DE, USA). RNA was converted to cDNA using the Applied Biosystems High Capacity cDNA Archive kit with RNase inhibitor at 50 U/sample (Applied Biosystems, Foster City, CA, USA). cDNA samples were stored at -20°C pending TaqMan^® ^assay.

The Jurkat and Daudi cell lines were seeded at a concentration of 4 × 10^5^/ml in RPMI-1640 medium (Sigma, Ayrshire, UK)) with 10% fetal bovine serum (Gibco, Invitrogen, Stockholm, Sweden) at 37°C. After 12 h, 1.6 × 10^6 ^cells were used for incubation with methotrexate for 24 h at 37°C. The same procedure as for cohort I was followed for the subsequent handling. Description of cell lines and methods for Western blot can be found in Additional file 3.

### Cytotoxicity measurement

The cytotoxic effect of methotrexate treatment of PBMCs and cell lines was estimated using a cytotoxicity detection kit (LDH; Roche, Mannheim, Germany). The procedure was performed according to manufacturer's recommendation.

### Quantitative PCR

For this study we used TaqMan assays from Applied Biosystems to interrogate the splice forms for *PTPN22*. At the time the study was done, the assays available were Hs00247352_m1 and Hs00249262_m1. Hs00247352_m1 is specific for the short splice form *PTPN22*_v4/LYP2, while the latter detects the long splice form PTNP22_v1/LYP1. This assay will also detect the recently reported splice form *PTPN22*_v3/LYP3 [13] as well as the recently updated Refseq isoform *PTPN22*_v2. We refer to these three splice forms, *PTPN22*_v1, *PTPN22*_v2 and *PTPN22*_v3, as *PTPN22*-long isoforms. Hs00206029_m1 was used for the endogenous reference gene *ZNF592*. *ZNF592 *was selected as a normalizer gene based on a survey of oligonucleotide array expression data for 44,928 transcripts across a compendium of 9,270 hybridizations, including multiple studies involving different types of cells and tissues. In this broad survey, *ZNF592 *had substantially less variability than other commonly utilized endogenous controls. Specifically, it had a coefficient of variation that was at the 0.01th percentile of variation among all surveyed transcripts (Table S2 in Additional file 2). Consistent with this finding, other groups have independently characterized *ZNF592 *as a ubiquitously expressed transcript in a panel of 24 adult human tissues by semi-quantitative PCR and northern blotting [18]. Expression measurements were made using the delta-delta relative quantification method according to the Applied Biosystems protocol. The mean delta Ct values for controls were used as calibrator. For cohort I each sample was measured six times, and for cohorts II and IV it was done in duplicates, and average values were calculated and used as relative measurements. For cohort III, RNA expression levels in human PBMCs were measured by using the ABI 7900HT Sequence detector (Sequence Detector Software 2.2.3) according to manufacturer's instructions. Relative quantification values were calculated from ΔΔCt values using the Sequence Detection System software, and further analyzed in a Spotfire-guided application (Spotfire Decision-Site 8™, TIBCO Software Inc. Somerville, MA, USA) developed within the Pfizer Massachusetts Research Business Technologies Department.

### Statistics

For comparing differences between groups, we used the non-parametric Mann-Whitney test and Kruskal-Wallis test with *P *= 0.05 as a threshold for significance with Bonferroni correction when necessary.

## Results

### Differential expression of *PTPN22 *splice forms in PBMCs from healthy controls and patients with RA

**Figure 1:**
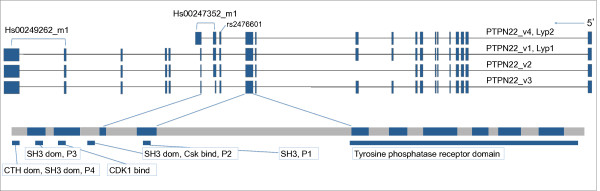
**Exonic-intronic structure of splice forms and domain representation of *PTPN22***. A genomic illustration of *PTPN22 *in human chromosome 1p32. Hs00247352_m1 and Hs00249262_m1 are assay numbers (Applied Biosystems). The four SRC homology 3 (SH3) domains are denoted by P1 to P4 and are typically involved in protein-protein interaction. Known binding sites for carboxy-terminal Src kinase (Csk) and CD2-binding protein 1 (CD2BP1) are located in P2 and P4 (carboxy-terminal homology (CTH) domain), respectively. The carboxyl terminus of Lyp1 contains a consensus motif for cyclin-dependent kinase 1 (CDK1). The exonic and basic domain structure is deduced from Ensembl (Ensembl 64, September 2011) [30] and UCSC Genome Browser (GRCh37/hg19 assembly) [31] and published articles [3,12].

To evaluate expression of the *PTPN22 *splice forms, we measured the expression levels of *PTPN22*-long isoforms and the short isoform *PTPN22*_v4 (LYP2; Figure 1), relative to *ZNF592 *expression in PBMCs of healthy individuals and RA patients (cohort I) after 6 h of cultivation of cells without stimulation. For both assays, we detected a difference in average expression between patients and controls. For *PTPN22*_v4 we saw a reduction in expression (0.85-fold decrease, non-significant) in RA cases and for *PTPN22*-long isoforms an increase in expression for RA cases (1.20-fold increase, *P *= 0.006) (Table 1).

**Table 1:** Expression of *PTPN22 *transcripts in peripheral blood mononuclear cells from cohorts I, II and III

	Median relative quantity values		
		
	Control group	RA patient group	*P*-value^a^
Cohort I	*n *= 44	*n *= 44	
*PTPN22*_v4	1.05	0.85	0.08
*PTPN22*-long	1.03	1.20	0.006
long/v4^b^	1.01	1.42	6 × 10^-9^
			
Cohort II	*n *= 19	*n *= 47	
*PTPN22*_v4	0.98	0.96	0.85
*PTPN22*-long	0.97	1.07	0.25
long/v4^b^	0.96	1.15	0.02
			
Cohort III	*n *= 48	*n *= 48	
*PTPN22*_v4	0.96	0.72	1.2 × 10^-4^
*PTPN22*-long	0.97	0.92	0.2
long/v4^b^	1.04	1.24	0.01

^a^*P*-value for comparison of rheumatoid arthritis (RA) patient group and control group, Mann-Whitney test. ^b^The ratio of *PTPN22*-long and *PTPN22*_v4 was calculated for each sample separately.

**Figure 2:**
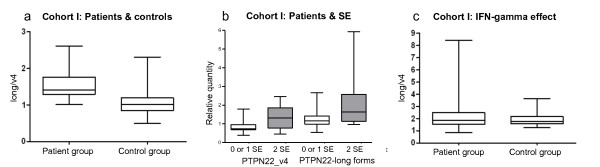
**Expression of *PTPN22 *splice forms**. **(a) **The ratio of *PTPN22*-long forms and *PTPN22*_v4 for rheumatoid arthritis (RA) patients and controls in cohort I. **(b) **Expression of *PTPN22*_v4 and *PTPN22*-long forms for RA patients stratified by copies of the shared epitope (SE), 0 or 1 (white boxes) versus 2 (grey boxes). **(c) **The effect of IFNγ *ex vivo *stimulation on expression ratio.

Since we used two assays to measure every sample, we could normalize the expression data by calculating a ratio. This reflects the balance between productions (and stability) of these splice forms for each individual and provides a better measure for comparing individuals by reducing the effect that the endogenous reference may have. By comparing the ratio of expression (Figure 2), we found that *PTPN22 *expression in RA cases is significantly different compared with that in controls (*P *= 6 × 10^-9^), which is a result of significantly higher expression of *PTPN22*-long isoforms and simultaneously slightly lower expression of *PTPN22*_v4 in patients (Table 1).

To replicate our findings, we measured expression of the *PTPN22 *splice forms in two independent collections of mRNA from whole blood from 19 controls and 47 RA patients from a Swedish population (cohort II) and 48 controls and 48 RA patients from the US (cohort III). The difference in expression was significant for the ratio of the transcripts (*P *= 0.02 and *P *= 0.01 for cohorts II and III, respectively) and demonstrated a similar trend for higher expression of *PTPN22*-long isoforms in cohort II and lower expression of *PTPN22*_v4 in cohort III (*P *= 1.2 × 10^-4^) for patients with RA (Table 1).

### IFNγ-induced expression of *PTPN22 *splice forms in healthy controls and patients with RA

To facilitate a mechanistic interpretation of differential expression of *PTPN22 *splice forms, we also measured the expression of these in IFN**γ-**stimulated PBMCs from the same healthy individuals and RA patients (cohort I). We found that the average expression of *PTPN22 *splice forms in these samples was similar for RA patients and controls for *PTPN22*_v4 and *PTPN22*-long forms, as well as for the ratio between the two (Table 2). These data suggest that proinflammatory conditions, such as IFN**γ **stimulation, may directly change the transcriptional profile for *PTPN22 *splice forms and that PBMCs from healthy individuals are able to produce higher amounts of *PTPN22*-long forms.

**Table 2:** Expression of *PTPN22 *transcripts in peripheral blood mononuclear cells from healthy controls and rheumatoid arthritis patients after stimulation with IFNγ (cohort I)

	Median relative quantity values		
		
	Control group	RA patient group	*P*-value^a^
Cohort I			
*PTPN22*_v4	0.71	0.68	NS
*PTPN22*-long	1.36	1.38	NS
long/v4	1.75	1.87	NS

All values are relative to the average expression of non-induced controls. ^a^*P*-value for comparison of the patient group and control group, Mann-Whitney test. NS, not significant.

### The influence of major RA risk factors on expression of *PTPN22 *splice forms in PBMCs of RA patients

We analyzed the expression data with regard to several parameters related to the risk of RA or disease activity, including gender, age at disease onset, rheumatoid factor (RF) and ACPA status, smoking, the *HLA-DRB1 *SE, polymorphisms in the *PTPN22 *locus, DAS28 at baseline and type of medication for cohort I.

The *HLA-DRB1 *SE was found to be moderately associated with the expression of *PTPN22*. As can be seen in Figure 2, RA patients carrying two copies of the SE had moderately higher expression of both *PTPN22*_v4 (*P *= 0.04) and *PTPN22*-long forms (*P *= 0.02) compared to individuals with no or only one copy. However, the ratio between expression values of the short and long forms does not significantly differ in individuals with two copies of the SE allele in comparison with those with one or no SE alleles.

The RA risk SNP rs2476601 (*PTPN22 *R620W) did not associate with the expression of splice forms in our study, nor did any other polymorphism in the *PTPN22 *locus show a consistent association. A list of all tested SNPs can be found in Table S3 Additional file 2.

### Influence of methotrexate on *PTPN22 *expression

**Figure 3:**
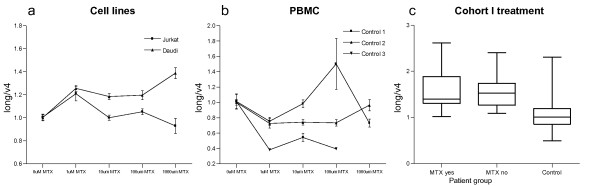
**Effect of methotrexate on expression of *PTPN22 *splice forms**. Investigating a possible effect of methotrexate (MTX) treatment. **(a,b) **For both cell lines (a) and peripheral blood mononuclear cells (PBMCs) (b), cells were incubated with medium and a range of concentrations of methotrexate for 24 h followed by quantitative PCR measurement of *PTPN22 *and cytotoxicity analysis (Figure S2 in Additional file 1). The ratio of *PTPN22*_long and *PTPN22*_v4 is shown in the graphs with standard error. **(c) **Boxplots showing the distribution in cohort I of the ratio of *PTPN22*_long and *PTPN22*_v4 expression stratified by methotrexate treatment. In cohort I, 23 patients were on methotrexate treatment at the time of sampling.

Since most of the RA patients were treated with a spectrum of anti-inflammatory drugs and disease-modifying antirheumatic drugs (DMARDs), we tested how these influence the expression of *PTPN22 *mRNA. The direct effect of pharmacological levels of methotrexate was investigated in two cell lines and in PBMCs from healthy donors (Figure 3a,b); as can be seen in the figures, we found only moderate effects on *PTPN22 *expression. Additionally, we compared the expression levels of *PTPN22 *between RA patients with and without methotrexate treatment at the time of blood donation and found no differences (Figure 3c). We also found no effect of type of other medications on the relative expression or the ratio of the splice forms (data not shown).

### Expression of *PTPN22 *splice forms in PBMCs from multiple sclerosis patients

To control for disease specificity of differential expression of *PTPN22 *splice forms, we analyzed independent collections of mRNA from PBMCs from individuals with MS. The MS patient group showed significantly higher expression of *PTPN22*_v4 compared to the control group (*P *= 0.01; same controls and same measurement as for cohort I) and consequently a lower ratio of splice forms compared to controls (Table 3). The same control group as for cohort I was used for this analysis.

**Table 3:** Expression of *PTPN22 *transcripts in peripheral blood mononuclear cells for a cohort of 60 multiple sclerosis patients (cohort IV)

	Median relative quantity values		
		
	Control group	MS Patient group	*P*-value^a^
*Cohort IV*			
*PTPN22*_v4	1.05	1.36	0.01
*PTPN22*-long	1.03	0.91	NS
long/v4	1.01	0.68	0.0003

All values are relative to the average expression of non-induced controls. Controls are from cohort I using the previous measurement. The same protocol for sample preparation was used as for cohort IV. ^a^*P*-value for comparison of the patient group and control group, Mann-Whitney test. NS, not significant.

## Discussion

Our major finding in the current study is that the expression of known splice forms of *PTPN22 *in PBMCs is different for RA patients and controls. The difference between patients and controls was even more pronounced for the ratio of the expression of splice forms, which may reflect a shift in the balance of products: the cells from RA patients had less Lyp2 (*PTPN22*_v4) and more Lyp1 (as represented by *PTPN22*-long forms) than cells from controls. These two splice forms are of special interest since it is known that they are translated at detectable levels [3], as also demonstrated in several cell lines (Figure S1 in Additional file 1). Our experimental findings are based on three independent cohorts and are contrasted by data from a cohort of patients with MS.

We did not find clear evidence of genotypic association with *PTPN22 *expression. The lack of association may be due to the cross-sectional design of the study or to non-optimal incubation times for PBMCs or a lack of statistical power. To test for a statistical difference for such regulation, we would need more data from individuals with the minor allele homozygotic state.

The association of *PTPN22 *expression with the *HLA-DRB1 *SE is interesting considering previous findings of interaction between these two RA risk factors [10,11]. This genetic interaction leads to an non-additive increase in risk for RA for individuals carrying any of the SE allele AND the PTPN22 risk allele. When we compared individuals having both or none of the susceptibility alleles, however, we did not find a significant effect on expression of *PTPN22 *(individuals homozygous or heterozygous for the minor allele of rs2476601 and positive for the SE versus individuals homozygous for major allele and no SE), but this comparison is also underpowered.

The observed correlation between the ratio of the splice forms and disease phenotype may have consequences since the known corresponding protein variants have pronounced structural differences. According to previous data [3,12], Lyp2 lacks several consensus sequences described for Lyp1 and other members of the PEST family of phosphatases, PTP-PEST (PTP with PEST domain, genomic designation *PTPN12*) and PTP-HSCF (PTP hematopoietic stem-cell fraction, genomic designation *PTPN18*). These differences might hint at a cause of functional divergence between the Lyp splice forms.

First, all three members of the PTP-PEST family contain a carboxy-terminal homology (CTH) domain, which is a binding site for CD2-binding protein 1 (CD2BP1), a known substrate for all three phosphatases within the PEST family [19]. CD2BP1 is expressed in T cells and has been proposed to be an important regulator of T-cell behavior by modulating CD2 activity, the latter being involved with the efficiency with which T-cell receptors bind a major histocompatibility complex (MHC) molecule with an antigenic peptide [20,21].

Second, it has also been reported that the carboxyl terminus of Lyp1 contains a consensus sequence recognized by cyclin-dependent kinase 1 (CDK1), a cell cycle regulatory kinase, suggesting that phosphorylation of Lyp1 may be cell cycle-dependent. The short variant of the protein, Lyp2 (corresponding to *PTPN22*_v4), lacks the consensus motif for CDK1, implying altered cell cycle-related regulation of this transcript in lymphoid cells compared to Lyp1 [3].

Third, the long variant of the protein, Lyp1, also contains four potential SRC homology 3 (SH3) domain binding sites, compared with a single motif in Lyp2, suggesting that the proteins may interact with different sets of SH3 domains [3]. For instance, Lyp2 does not contain the consensus motif PPPLPERTP, responsible in mice for binding p50csk (proto-oncogene c-Src in humans), as demonstrated in hematopoietic cells with both PEP and PTP-PEST [22,23]. c-Src is known to negatively regulate leukocyte-specific protein tyrosine kinase - an important mediator in cytokine production signaling [24]. In a study where similar proline-rich substrate-binding motifs were deleted in the carboxyl terminus of PTP-PEST, it was demonstrated that some of these motifs are required in order for the phosphatase to be able to inhibit B-cell activation [25].

It was previously suggested that the R620W variant of PTPN22 alters the protein-protein interaction with **c-Src tyrosine kinase**, CSK, with the subsequent down regulation of **lymphocyte-specific protein tyrosine kinase**, Lck, which is a gain of function of phosphatase [5]. Our data suggest that the structural background for T-cell activation could also be due to a shift in the expression of splice forms. Individuals with RA in the studied cohorts may generally have more of the longer and putatively more active *PTPN22 *transcripts, as represented by measurement of the 3' end of long splice forms, while healthy controls in our study have less. Further investigation is required to elucidate the disease-associated expression patterns of the individual long splice forms.

It remains unclear how the risk allele relates to expression of *PTPN22 *splice forms, although due to extensive linkage disequilibrium in the *PTPN22 *locus, any regulatory variant for alternative splicing could be of importance and should be investigated. However, our search for potential variants of splicing motifs within the *PTPN22 *gene did not reveal any clear candidate SNPs for mechanistic studies.

These possible functional differences between Lyp1 and Lyp2 were underappreciated previously and may suggest that a shift in the balance of splice forms could have a considerable impact on the inflammatory signaling. This difference in expression seems not to be significantly regulated by allelic variants and although it cannot be totally excluded according to our cell line experiments, the expression seems not to be affected by medication of the patients. Also, in the presence of a strong inflammatory environment (IFNγ in our study) the balance in transcript expression could be well compensated. Interestingly, our study of *PTPN22 *splice form expression in MS patients did not show a similar effect, which is in line with the fact that this disease is not associated with polymorphisms in the *PTPN22 *gene and supports disease specificity [2,26-29]. To resolve if this difference in expression is related to genetic association with *PTPN22 *variants, expression of *PTPN22 *splice forms in systemic lupus erythematosus and type 1 diabetes should be tested in a similar way.

## Conclusions

We found important differences in the expression of *PTPN22 *splice forms between healthy individuals and RA patients, which may increase a gain of function that influences development of the RA.

## Abbreviations

ACPA: anti-citrullinated protein antibody; IFN: interferon; LYP: lymphoid tyrosine phosphatase; MS: multiple sclerosis; NCBI: National Center for Biotechnology Information; PBMC: peripheral blood mononuclear cell; PBS: phosphate-buffered saline; PEST: proline-, glutamic acid-, serine-, and threonine-rich; RA: rheumatoid arthritis; RF: rheumatoid factor; SE: shared epitope.

## Competing interests

No personal financial interests are declared. The following authors have positions with Pfizer Research: Yongjing Guo, Andrew Hill, Padmalatha S Reddy, James Clark, Lih-Ling Lin and Margot O'Toole. Marcus Ronninger, Klementy Shchetynsky, Mohsen Khademi, Tomas Olsson, Maria Seddighzadeh and Leonid Padyukov have no potential conflicts of interest.

## Authors' contributions

MR performed the experiments on cohorts I and II, analysis and interpretation of data and drafted the manuscript. YG performed experiments and data analyses for cohort III, and revised the manuscript. KS performed the experiments on cohorts I and II, and drafted the manuscript. AH performed statistical and other data analyses for cohort III. MK collected material for cohort I. TO critically revised the manuscript. PSR did the sequence analysis of the various *PTPN22 *splice forms. MS performed genotyping of cohort I and revised the manuscript. JD made substantial contributions to the design of the study and critically revised the manuscript. LL critically revised the manuscript. MOT designed the cohort III study in which the original observation of variant differences associated with disease was made and critically revised the manuscript. LP designed study, interpreted data and drafted the manuscript. All authors have read and approved the manuscript for publication.

## Supplementary Material

Additional file 1 **Figures S1 and S2**. Figure S1: a western blot for PTPN22. Figure S2: graphs showing the cytotoxicity for methotrexate treatment of cell lines and PBMCs from healthy controls. Click here for file

Additional file 2 **Tables S1 to S3**. Table S1: study population composition. Table S2: identification of reference genes for quantitative PCR. Table S3: the SNPs used in the analysis of cohort I. Click here for file

Additional file 3 **Supplementary material and methods for western blotting**. Click here for file

## References

[B1] Begovich AB Carlton VE Honigberg LA Schrodi SJ Chokkalingam AP Alexander HC Ardlie KG Huang Q Smith AM Spoerke JM Conn MT Chang M Chang SY Saiki RK Catanese JJ Leong DU Garcia VE McAllister LB Jeffery DA Lee AT Batliwalla F Remmers E Criswell LA Seldin MF Kastner DL Amos CI Sninsky JJ Gregersen PK A missense single-nucleotide polymorphism in a gene encoding a protein tyrosine phosphatase (PTPN22) is associated with rheumatoid arthritis. Am J Hum Genet 2004 75 330 337 10.1086/422827 15208781 PMC1216068

[B2] Criswell LA Pfeiffer KA Lum RF Gonzales B Novitzke J Kern M Moser KL Begovich AB Carlton VE Li W Lee AT Ortmann W Behrens TW Gregersen PK Analysis of families in the multiple autoimmune disease genetics consortium (MADGC) collection: the PTPN22 620W allele associates with multiple autoimmune phenotypes. Am J Hum Genet 2005 76 561 571 10.1086/429096 15719322 PMC1199294

[B3] Cohen S Dadi H Shaoul E Sharfe N Roifman CM Cloning and characterization of a lymphoid-specific, inducible human protein tyrosine phosphatase, Lyp. Blood 1999 93 2013 2024 10068674

[B4] Rieck M Arechiga A Onengut-Gumuscu S Greenbaum C Concannon P Buckner JH Genetic variation in PTPN22 corresponds to altered function of T and B lymphocytes. J Immunol 2007 179 4704 4710 17878369 10.4049/jimmunol.179.7.4704

[B5] Vang T Congia M Macis MD Musumeci L Orru V Zavattari P Nika K Tautz L Tasken K Cucca F Mustelin T Bottini N Autoimmune-associated lymphoid tyrosine phosphatase is a gain-of-function variant. Nat Genet 2005 37 1317 1319 10.1038/ng1673 16273109

[B6] Arechiga AF Habib T He Y Zhang X Zhang ZY Funk A Buckner JH Cutting edge: the PTPN22 allelic variant associated with autoimmunity impairs B cell signaling. J Immunol 2009 182 3343 3347 10.4049/jimmunol.0713370 19265110 PMC2797545

[B7] Carlton VE Hu X Chokkalingam AP Schrodi SJ Brandon R Alexander HC Chang M Catanese JJ Leong DU Ardlie KG Kastner DL Seldin MF Criswell LA Gregersen PK Beasley E Thomson G Amos CI Begovich AB PTPN22 genetic variation: evidence for multiple variants associated with rheumatoid arthritis. Am J Hum Genet 2005 77 567 581 10.1086/468189 16175503 PMC1275606

[B8] Wan Taib WR Smyth DJ Merriman ME Dalbeth N Gow PJ Harrison AA Highton J Jones PB Stamp L Steer S Todd JA Merriman TR The PTPN22 locus and rheumatoid arthritis: no evidence for an effect on risk independent of Arg620Trp. PLoS One 2010 5 e13544 10.1371/journal.pone.0013544 20975833 PMC2958827

[B9] Rodriguez-Rodriguez L Taib WR Topless R Steer S Gonzalez-Escribano MF Balsa A Pascual-Salcedo D Gonzalez-Gay MA Raya E Fernandez-Gutierrez B Gonzalez-Alvaro I Bottini N Witte T Viken MK Coenen MJ van Riel PL Franke B den Heijer M Radstake TR Wordsworth P Lie BA Merriman TR Martin J The PTPN22 R263Q polymorphism is a risk factor for rheumatoid arthritis in Caucasian case-control samples. Arthritis Rheum 2011 63 365 372 10.1002/art.30145 21279993

[B10] Kallberg H Padyukov L Plenge RM Ronnelid J Gregersen PK van der Helm-van Mil AH Toes RE Huizinga TW Klareskog L Alfredsson L Gene-gene and gene-environment interactions involving HLA-DRB1, PTPN22, and smoking in two subsets of rheumatoid arthritis. Am J Hum Genet 2007 80 867 875 10.1086/516736 17436241 PMC1852748

[B11] Morgan AW Thomson W Martin SG Carter AM Erlich HA Barton A Hocking L Reid DM Harrison P Wordsworth P Steer S Worthington J Emery P Wilson AG Barrett JH Reevaluation of the interaction between HLA-DRB1 shared epitope alleles, PTPN22, and smoking in determining susceptibility to autoantibody-positive and autoantibody-negative rheumatoid arthritis in a large UK Caucasian population. Arthritis Rheum 2009 60 2565 2576 10.1002/art.24752 19714585

[B12] Vang T Miletic AV Arimura Y Tautz L Rickert RC Mustelin T Protein tyrosine phosphatases in autoimmunity. Annu Rev Immunol 2008 26 29 55 10.1146/annurev.immunol.26.021607.090418 18303998

[B13] Wang S Dong H Han J Ho WT Fu X Zhao ZJ Identification of a variant form of tyrosine phosphatase LYP. BMC Mol Biol 2010 11 78 10.1186/1471-2199-11-78 21044313 PMC2987843

[B14] Arnett FC Edworthy SM Bloch DA McShane DJ Fries JF Cooper NS Healey LA Kaplan SR Liang MH Luthra HS The American Rheumatism Association 1987 revised criteria for the classification of rheumatoid arthritis. Arthritis Rheum 1988 31 315 324 10.1002/art.1780310302 3358796

[B15] Plenge RM Seielstad M Padyukov L Lee AT Remmers EF Ding B Liew A Khalili H Chandrasekaran A Davies LR Li W Tan AK Bonnard C Ong RT Thalamuthu A Pettersson S Liu C Tian C Chen WV Carulli JP Beckman EM Altshuler D Alfredsson L Criswell LA Amos CI Seldin MF Kastner DL Klareskog L Gregersen PK TRAF1-C5 as a risk locus for rheumatoid arthritis - a genomewide study. N Engl J Med 2007 357 1199 1209 10.1056/NEJMoa073491 17804836 PMC2636867

[B16] Lundstrom E Kallberg H Alfredsson L Klareskog L Padyukov L Gene-environment interaction between the DRB1 shared epitope and smoking in the risk of anti-citrullinated protein antibody-positive rheumatoid arthritis: all alleles are important. Arthritis Rheum 2009 60 1597 1603 10.1002/art.24572 19479873 PMC2732897

[B17] Swanberg M Lidman O Padyukov L Eriksson P Akesson E Jagodic M Lobell A Khademi M Borjesson O Lindgren CM Lundman P Brookes AJ Kere J Luthman H Alfredsson L Hillert J Klareskog L Hamsten A Piehl F Olsson T MHC2TA is associated with differential MHC molecule expression and susceptibility to rheumatoid arthritis, multiple sclerosis and myocardial infarction. Nat Genet 2005 37 486 494 10.1038/ng1544 15821736

[B18] Nicolas E Poitelon Y Chouery E Salem N Levy N Megarbane A Delague V CAMOS, a nonprogressive, autosomal recessive, congenital cerebellar ataxia, is caused by a mutant zinc-finger protein, ZNF592. Eur J Hum Genet 2010 18 1107 1113 10.1038/ejhg.2010.82 20531441 PMC2987462

[B19] Spencer S Dowbenko D Cheng J Li W Brush J Utzig S Simanis V Lasky LA PSTPIP: a tyrosine phosphorylated cleavage furrow-associated protein that is a substrate for a PEST tyrosine phosphatase. J Cell Biol 1997 138 845 860 10.1083/jcb.138.4.845 9265651 PMC2138048

[B20] Bierer BE Burakoff SJ T-lymphocyte activation: the biology and function of CD2 and CD4. Immunol Rev 1989 111 267 294 10.1111/j.1600-065X.1989.tb00549.x 2483402

[B21] Selvaraj P Plunkett ML Dustin M Sanders ME Shaw S Springer TA The T lymphocyte glycoprotein CD2 binds the cell surface ligand LFA-3. Nature 1987 326 400 403 10.1038/326400a0 2951597

[B22] Davidson D Cloutier JF Gregorieff A Veillette A Inhibitory tyrosine protein kinase p50csk is associated with protein-tyrosine phosphatase PTP-PEST in hemopoietic and non-hemopoietic cells. J Biol Chem 1997 272 23455 23462 10.1074/jbc.272.37.23455 9287362

[B23] Cloutier JF Veillette A Association of inhibitory tyrosine protein kinase p50csk with protein tyrosine phosphatase PEP in T cells and other hemopoietic cells. EMBO J 1996 15 4909 4918 8890164 PMC452228

[B24] Mustelin T Tasken K Positive and negative regulation of T-cell activation through kinases and phosphatases. Biochem J 2003 371 15 27 10.1042/BJ20021637 12485116 PMC1223257

[B25] Davidson D Veillette A PTP-PEST, a scaffold protein tyrosine phosphatase, negatively regulates lymphocyte activation by targeting a unique set of substrates. EMBO J 2001 20 3414 3426 10.1093/emboj/20.13.3414 11432829 PMC125513

[B26] Begovich AB Caillier SJ Alexander HC Penko JM Hauser SL Barcellos LF Oksenberg JR The R620W polymorphism of the protein tyrosine phosphatase PTPN22 is not associated with multiple sclerosis. Am J Hum Genet 2005 76 184 187 10.1086/427244 15580548 PMC1196423

[B27] Matesanz F Rueda B Orozco G Fernandez O Leyva L Alcina A Martin J Protein tyrosine phosphatase gene (PTPN22) polymorphism in multiple sclerosis. J Neurol 2005 252 994 995 10.1007/s00415-005-0795-y 15765267

[B28] Hinks A Barton A John S Bruce I Hawkins C Griffiths CE Donn R Thomson W Silman A Worthington J Association between the PTPN22 gene and rheumatoid arthritis and juvenile idiopathic arthritis in a UK population: further support that PTPN22 is an autoimmunity gene. Arthritis Rheum 2005 52 1694 1699 10.1002/art.21049 15934099

[B29] De Jager PL Sawcer S Waliszewska A Farwell L Wild G Cohen A Langelier D Bitton A Compston A Hafler DA Rioux JD Evaluating the role of the 620W allele of protein tyrosine phosphatase PTPN22 in Crohn's disease and multiple sclerosis. Eur J Hum Genet 2006 14 317 321 10.1038/sj.ejhg.5201548 16391555

[B30] Ensembl. http://www.ensembl.org

[B31] UCSC Genome Browser. http://genome.ucsc.edu/

