# Supplementary material for: The balance of expression of PTPN22 splice forms is significantly different in rheumatoid arthritis patients compared with controls
Source: Genome Med. 2012 Jan 20;4(1):2. doi: 10.1186/gm301 (PMC3334550; doi:10.1186/gm301)
Supplement: Additional file 1 — Figures S1 and S2. Figure S1: a western blot for PTPN22. Figure S2: graphs showing the cytotoxicity for methotrexate treatment of cell lines and PBMCs from healthy controls. [file gm301-S1.PDF]

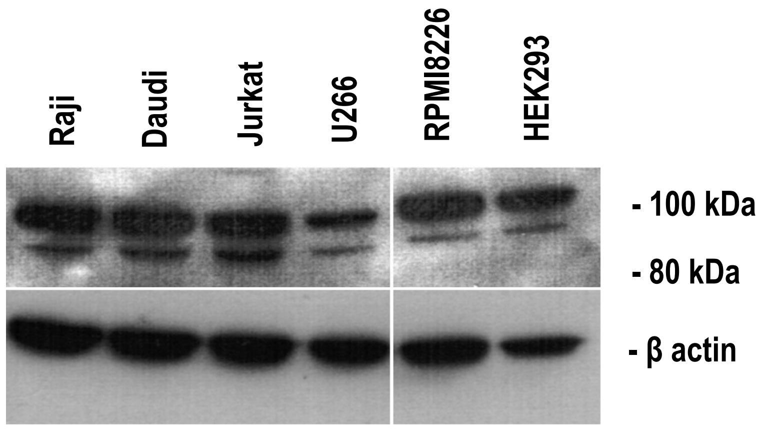

Figure S1. Expression of Lyp1 and Lyp2 in human lymphatic cell lines and embrionic kidney cell line. All cell lysates were separated by 4-12% PAGE and incubated with PTP22 (T-16) antibody for Western blotting. Products of sizes corresponding to Lyp1/2 were detected in cell-lines representing human T-lymphocyte (Jurkat), B-lymphocyte (Raji, RPMI8226) and B-lymphoblast (Daudi, U266) populations, as well as human embryonic kidney cell line (HEK293). β-actin expression was detected for loading control. See additional file 4 for material and methods for Western blotting.

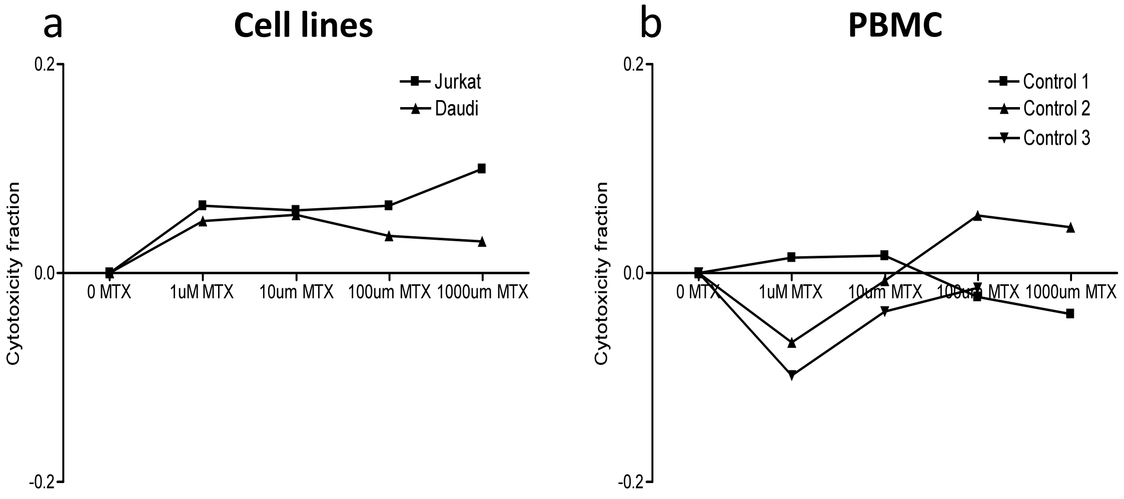

Figure S2. Cytotoxicity measurement for methotrexate treated cells. Data for the cytotoxicity effect by methotrexate in the cell treatment experiments is shown in a) and b). The amount of cytotoxicity is represented as increased fraction of lactate dehydrogenase release compared to non-treated cells.
