# Supplementary material for: The balance of expression of PTPN22 splice forms is significantly different in rheumatoid arthritis patients compared with controls
Source: Genome Med. 2012 Jan 20;4(1):2. doi: 10.1186/gm301 (PMC3334550; doi:10.1186/gm301)
Supplement: Additional file 2 — Tables S1 to S3. Table S1: study population composition. Table S2: identification of reference genes for quantitative PCR. Table S3: the SNPs used in the analysis of cohort I. [file gm301-S2.DOC]

Table S1 - Study population composition

|  | |  |  |  |  |
| --- | --- | --- | --- | --- | --- |
|  | Female | Male | Average Age | Av. Age Female | Av. Age Male |
| Cohort I RA |  |  |  |  |  |
| Patients | 36 | 8 | 56.2 | 55.5 | 59.5 |
| Controls | 36 | 8 | 46.5 | 46.2 | 47.9 |
| Cohort II RA |  |  |  |  |  |
| Patients | 39 | 8 | 52.6 | 51.7 | 56.8 |
| Controls | 19 | 3 | 54.1 | 53.5 | 57.7 |
| Cohort III RA |  |  |  |  |  |
| Patients | 35 | 13 | 57.8 | 57.6 | 58.0 |
| Controls | 34 | 14 | 57.4 | 58.3 | 55.1 |
| Cohort IV MS |  |  |  |  |  |
| Patients | 37 | 23 | 39.9 | 39.2 | 41.0 |

Table S2 – Identification of reference gene for quantitative real-time PCR ^a^.

| PROBESET | PERCENTILE5 | MEDIAN | PERCENTILE95 | CV | Gene Title | Gene Symbol | NCBI Gene ID |
| --- | --- | --- | --- | --- | --- | --- | --- |
| 226042_at | 313 | 443 | 607 | 0.211702 | hypothetical protein FLJ21128 | FLJ21128 | 80153 |
| 229356_x_at | 362 | 497 | 704 | 0.21305 | homolog of yeast INO80 | INO80 | 54617 |
| 222661_at | 243 | 357 | 502 | 0.222145 | angiogenic factor VG5Q | VG5Q | 55109 |
| 242621_at | 160 | 233 | 336 | 0.227672 | zinc finger protein 498 | ZNF498 | 221785 |
| 227507_at | 253 | 370 | 528 | 0.229847 | Zinc finger protein 592 | ZNF592 | 9640 |

^a^ A database of transcript expression levels across 9,270 hybridizations of the Affymetrix HG-U133A and B array designs (comprising 44,928 probesets) was used to assess potential endogenous control transcripts. Samples in this dataset included a broad range of diverse human tissues and cell lines. Each probeset was characterized by its 5^th^, 50^th^ (median), and 95^th^ percentile expression level (by Affymetrix MAS5 signal algorithm), and coefficient of variation (CV). Probesets were ranked from least variable to most variable by CV. The table shows the 5 probesets with lowest CV.  Our selected endogenous control, ZNF592, ranked 5^th^ of 44,928 probesets (0.01th percentile). It was broadly and stably expressed (expression signal range 253-528, CV = 23%). For comparison, other common endogenous control transcripts such as GAPDH and Actin were found to be highly expressed, but more variable than ZNF592 in this dataset.  Specifically, probesets for GAPDH (NCBI Gene ID 2597) had median expression levels from 2905-10050 and CVs ranging from 46-69%, and beta actin (NCBI Gene ID 60) probesets had median exprsession levels from 2767-11949 and CVs from 41-65%.

Table S3 - List of SNPs used in study of cohort I.

| SNP rs id | Position chr 1 |
| --- | --- |
| rs2040041 | 114114146 |
| rs12117799 | 114120127 |
| rs2797409 | 114131725 |
| rs3827733 | 114140112 |
| rs3811021 | 114158186 |
| rs2476599 | 114164982 |
| rs3789607 | 114167957 |
| rs2476601 | 114179091 |
| rs1217407 | 114195271 |
| rs1217418 | 114202754 |
| rs6665194 | 114219366 |
| rs12566340 | 114221851 |
| rs7529353 | 114221985 |
| rs1217394 | 114235182 |
| rs10745340 | 114238493 |
| rs1217401 | 114240474 |
| rs17464525 | 114245422 |
| rs971173 | 114249437 |
| rs1217390 | 114252830 |
| rs878129 | 114260718 |
| rs11811771 | 114264572 |
| rs11102703 | 114267633 |
| rs7545038 | 114280953 |
| rs1503832 | 114291212 |
| rs11485101 | 114303491 |
