# Supplementary material for: The balance of expression of PTPN22 splice forms is significantly different in rheumatoid arthritis patients compared with controls
Source: Genome Med. 2012 Jan 20;4(1):2. doi: 10.1186/gm301 (PMC3334550; doi:10.1186/gm301)
Supplement: Additional file 3 — Supplementary material and methods for western blotting. [file gm301-S3.DOC]

Supplementary Material and Methods

Western blot. All cells were seeded 12 hours before lysis at a concentration 2*10^5^ .Cell lysates were prepared in 50 mM Tris–HCl, pH 7.4, 1% NP40, 150 mM NaCl, 1 mM EDTA, 1% glycerol and protease inhibitor cocktail (Roche Diagnostics GmbH, Mannheim, Germany). 30 μg of proteins were separated by 4%–12% PAGE and transferred to PVDF-membranes using the iBlot® dry transfer system (Invitrogen by Life Technologies, Sweden). The membranes were blocked in 5% milk in TBS-Tween20, followed by incubation with primary antibody for 2 h, and a secondary antibody for 1 h at room temperature. The PVDF membranes were developed using Western Lightning–ECL from PerkinElmer, Sweden AB. Images were obtained with a LAS-1000 from Fujifilm. Antibodies: goat polyclonal IgG PTP22 (T-16) primary antibody (sc-48922) – dilution 1:500, donkey anti-goat IgG-HRP secondary antibody (sc-2020) – dilution 1:2500, (SantaCruz Biotechnologies, Europe), β-actin (45 kD) (Sigma-Aldrich, Sweden), anti-biotin, HRP-linked antibody (Cell Signaling Technology®, In Vitro AB, Sweden). Molecular weight standards: Biotinylated Protein Ladder (Cell Signaling Technology®, In Vitro AB, Sweden), Spectra ™ Multicolor Broad Range Protein Ladder (Fermentas, Thermo+Fisher Scientific, Sweden).

Cell lines for protein expression. Human lymphiod cell lines Jurkat, Daudi, Raji, U266-1984 (U266), RPMI8226, and embryonic kidney cell-line HEK293 (ATCC Collection, USA) were cultured in 5% CO_2_ at 37 °C in RPMI1640 (Sigma, Steinhem, Germany) and Dulbecco’s Modified Eagle Medium respectively, supplemented with 10% heat-inactivated fetal bovine serum, 2 mM glutamine, 50 μg/ml of streptomycin and 50 μg/ml of penicillin (Gibco, Invitrogen, Sweden).
